# Genetically Engineered Porcine Organs for Human Xenotransplantation

**DOI:** 10.7759/cureus.29089

**Published:** 2022-09-12

**Authors:** Maryam Shahab, Nihal Ud Din, Nimra Shahab

**Affiliations:** 1 Internal Medicine, Queens Internal Medicine and Geriatrics, New York, USA; 2 Internal Medicine, Nowshera Medical College, Nowshera, PAK

**Keywords:** xenograft survival, xenotransplantation, xenoantigens, transplant rejection, porcine, perv, genetic modification, crispr/cas9

## Abstract

Xenotransplantation holds a promising future for many patients, especially those with end-stage renal disease or uncontrollable serum glucose levels. Porcine organs are viewed as the perfect candidate for a source of xenografts. However, the recipient's immunity, incompatibility of biologic systems, and transfer of new pathogenic organisms are all obstacles to clinical xenotransplantation, in addition to the risk of zoonosis and xenoantigens. Genetic modification of pigs using clustered regularly interspaced short palindromic repeat (CRISPR)-CRISPR-associated protein 9 (Cas9) resulted in the production of porcine endogenous retrovirus (PERV)-free offsprings with the consequent removal of many clinical complications post-transplantation. Such as minimizing both acute and chronic inflammation, in addition to suppressing rejection reactions, which may prolong graft survival. To build on these recent successes, it is important to look at the limits of genetic engineering and develop ways to advance the field of xenotransplantation and reverse xenotransplantation clinical applications forward. Still, significant problems remain with clinical human xenotransplantation; future work should focus on developing an ideal genetically engineered swine donor source that can improve long-term graft survival and suppress the immune system in a clinically useful way.

## Introduction and background

It has been more than a century since researchers began investigating the possibility of using animal tissues and organs in humans. Xenotransplantation has risen in popularity as a result of the success of allotransplantation [1], which is ‘the transplantation of an organ or tissue from one individual to another of the same species with a different genotype’ [2], as a treatment for a variety of illnesses, including organ failure. Xenotransplantation is ‘the deliberate transfer of living cells, tissues, or organs from individuals of one species to individuals of another’ [3]. It is believed that using pigs as a source of transplants will allow transplantation to have a significantly greater impact on medicine and public health than allotransplantation [4]. However, this has not yet been proven. It is generally agreed that the recipient's immunity, incompatibility of biologic systems, and transfer of new pathogenic organisms are all biologic obstacles to xenotransplantation that are currently impeding its use in clinical settings [4-9], in addition to the ethical concerns pertaining to xenotransplantation [4,10]. The genetic modification of animal sources could be a viable answer to the xenotransplantation problem [3-11]. The tools for genetically altering animals have progressed significantly over the past two decades [12]. The advancement of reversal xenografts, in which human stem cells are implanted into pigs under conditions that promote differentiation and enlargement into functioning tissues and maybe organs, has also been aided by the advancement of this novel type of xenograft [3]. It is necessary to evaluate the constraints of genetic engineering and to propose models for advancing xenotransplantation and reverse xenotransplantation clinical applications in order to build on these recent successes.

## Review

Porcine organs as a source for xenotransplantation 

Porcine organs are attractive candidates in multiple studies due to their close resemblance in anatomic size and physiologic function to human organs [1,3-9]. In addition, pigs are easy to breed and produce a large number of offspring [1,3-10], grow into adult size within six months [6,9,10], and are readily available [4,7,10]. Although porcine is seen as a promising source for xenotransplantation [7,9], immunological incompatibility [1,3-9,11], and the possibility of zoonotic infections with micro-organisms have limited the therapeutic use of swine organs. The most striking example being porcine endogenous retrovirus (PERV) [3-5,7-9], a gamma retrovirus, as it may induce a state of immunosuppression and carcinogenesis [5-8]. In addition to PERV, other zoonotic organisms are capable of being transmitted during a xenotransplant, including herpes virus, cytomegalovirus, human immunodeficiency virus, rabies virus, and Epstein-Barr virus [7-9].

Based on cell tropism, sequence variation, or receptor interference, three subtypes of PERV have been designated as PERV-A, PERV-B, or PERV-C, respectively [7]. PERV-A and PERV-B are found in the genetic composition of all swine, whilst PERV-C isn’t present in all pig genomes [7,8]. Human cells may be infected by PERV-A and PERV-B [8], constituting a xenotransplantation danger. However, PERV-C only infects pig cells [5,7]. Successful production of PERV-inactivation and resultant PERV-inactivated pigs may be used for therapeutic xenotransplantation in preventing cross-species viral vertical transmission [5-9,11]. DNA-damage-induced senescence or apoptosis was thought to be triggered by clustered regularly interspaced short palindromic repeats (CRISPR) and CRISPR-associated protein 9 (Cas9) cleavages of several PERV sites in the primary porcine fetal fibroblast cell line (FFF3) at the same time [5]. The deletion of all 62 copies of PERV in the FFF3 genome of a pig cell line resulted in the inactivation of PERV [5,7,9]. Somatic cell nuclear transfer (SCNT) of tailored fibroblasts from the main pig fibroblast cell line was utilized to create embryos, which were subsequently transferred into surrogates [5,6,11]. It was possible to create 100% PERV-free embryos, fetuses, and pigs only with the combined use of CRISPR/Cas9, p53 apoptosis inhibitor, and growth factor [5].

Porcine renal xenotransplantation

Transplantation of the pancreatic islets has recently been shown to be a viable therapy for patients with type 1 diabetes mellitus with unpredictable blood glucose levels and hypoglycemic events [11,13]. However, in order to improve the quality of life and survival, patients on chronic dialysis with end-stage renal disease are best treated with kidney transplantation [6,14]. There are more than 100,000 candidates on a waiting list for a renal transplant, of which 20% of the population never have the opportunity to obtain a transplant [6,7,14]. Identification of the ideal human candidate is very crucial for xenotransplantation, whether it be a renal or islet xenograft, as there are a limited number of donors for xenotransplant organs and countless number of patients waiting to prolong survival [9]. As a means of preventing T-cell rejection, suitable donor-recipient combinations are found using histocompatibility testing and pharmacological immunosuppression [3,6,14].

However, xenotransplantation has not yet been successful due to humans containing pre-existing antibodies that attach to porcine surface proteins and produce early antibody-mediated rejection (AMR) [6,8] and hyperacute rejection (HAR) [3,4,7,9,11], with consequent graft failure [14]. Eradication of xenoreactive antibodies resulted in the production of α-1,3-galactosyltransferase (GGTA1) knockout (KO) pigs while abolishing 70-90% of xenoantigens [6,15]. AMR of a renal transplant in GGTA1 KO pigs occurred within 6-17 days, even after treatment with multiple immunosuppressants, including rabbit anti-thymocyte globulin, tacrolimus, mycophenolic acid, and steroids [6]. Sid's blood group antigen (Sd_a_), a glycan antigen that is xenoreactive for both humans and monkeys, was discovered during the hunt for new xenoantigens that may serve as targets for future genetic alteration in donor pigs [6,11]. The β-1,4 N-acetylgalactosaminyltransferase (B4GALNT2) enzyme, which is found in pigs but not in humans, produces Sd_a_ [6,9,11]. The N-glycolylneuraminic acid, generated by the cytidine monophosphate-N-acetylneuraminic acid hydroxylase (CMAH) gene, is another xenoantigen in humans [6].

Multiplex genome editing in the pig has become feasible because of nuclease-based genome editing [6-9,11,15], which allows for the deletion of several genes in a solo reaction. In a study conducted by Adams et al., it was determined if xenoantigen-reduced pig kidneys with no human complement regulation genes might be transplanted into Rhesus monkeys to attain increased longevity utilizing pharmacological immunosuppression [6]. Once again, CRISPR/Cas9 was employed to remove three genes that encode enzymes involved in the synthesis of glycan xenoantigens: α-gal (GGTA1), N-glycolylneuraminic acid (CMAH), and Sd_a_ antigen (B4GALNT2) producing PERV-free pigs [5,11,14-16]. As pigs mature into an adult, surface α-gal antigens decline [9]. With a combination of T-cell depletion and therapeutic immunosuppression, GGTA1 KO/CD55 transgenic porcine kidneys can enable extended life by more than 300 days [3]. Yet still, graft failure was due to the results of AMR, implying that xenoreactive antibodies remained the major setback in the survival of a xenotransplant [6].

The genes GGTA1 and B4GALNT2 are essential for the synthesis of the xenoantigens α-gal and Sd_a_, which are engaged in the humoral rejection of xenografts [9]. Fetal fibroblasts were injected with plasmids containing the Cas9 endonuclease as well as gRNA targeting the GGTA1 and B4GALNT2 genes. Twenty-eight embryonic transplants led to 22 pregnancies, resulting in a fertility rate of 79%; 13 of the 22 pregnancies were brought to term, resulting in a total of 48 piglets, 40 of which survived with a viability of 83% [6]. Both α-gal and Sd_a_ were entirely absent in the GGTA1/B4GALNT2 KO pig kidney.

Porcine islet cell xenotransplantation 

Porcine islet cell xenotransplantation is looked upon as a promising source for patients diagnosed with type 1 diabetes mellitus [7-9,13,16]. Pig insulin is physiologically similar to human insulin [7,8]; in addition, insulin in swine is structurally identical to human insulin, with the exception of one amino acid at position B30, containing alanine and threonine in pigs and humans, respectively [9,11,16]. Attempts are being made to create genetically engineered swine that produce human insulin [11,16,17]. Previous research, conducted by Yang et al., utilized SCNT technology with nuclear transcription factor activator-like CRISPR/Cas9, paired with single-stranded oligonucleotides, to effectively create pigs that produced human insulin [18]. Even though the genetically modified swine produced human insulin, yet still had a distinct amino acid composition and structure. CRISPR/Cas9 technology was reportedly employed to create insulin-deficient pigs, whereas SCNT was used to create piglets that produced both insulin and human C-peptide [17].

Two markers of inflammation, among many others, have proven to signify the degree of inflammation and healing: interleukin-6 (IL-6) and C-reactive protein (CRP) [19]. IL-6 is an inflammatory mediator that has properties that both aid in inflammation [20] and assist in the resolution of inflammation [19,21], as well as having a variety of biological impacts on the inflammatory response [22], immune defense [21], and blood cell regeneration [20]. IL-6 receptor activity itself is implicated in the rise of IL-6 levels and the lowering of CRP levels in the serum [13]. By dampening the inflammatory process, antagonistic activity against the IL-6 receptor produces favorable outcomes for xenotransplantation and revascularization [22]. The long-term survival of grafted islet cells is dependent on islet revascularization [9,13,16]. For several days post-transplantation, pancreatic islet cell grafts are isolated from their blood supply and rely exclusively on diffusion for circulation and nutrients [9,13]. The focus of recent data by Min et al. was to determine if antagonistic IL-6 signaling activity post-transplantation of pancreatic islets has a substantial positive or negative effect on overall perfusion of islet grafts, whether the endothelial cells in the islet transplants originated totally from the porcine recipient, and its ultimate prognosis [13]. Tocilizumab, an IL-6R blocker [22], was used to minimize an acute inflammatory response following islet transplantation [11,13]. The tocilizumab-treated group showed significantly decreased levels of CRP [11]. Immunohistochemical studies revealed that IL-6 receptor inhibition substantially decreased the reperfusion of the transplanted islet [13]. As a result, the positive impact of blocking IL-6 receptors would be outweighed by the detrimental impact on islet revascularization. In general, the survival of the transplant was not increased by IL-6 receptor blockade. However, insulin from the transplanted islet kept blood glucose levels in the normal range [13,14].

Anti-CD40 and anti-CD154 monoclonal antibodies are regarded to have tremendous immunosuppressive capability by disrupting a crucial signaling cascade essential for proper activation stimulation of T-cells, B-cells, macrophages, and dendritic cells [11]. In recent data, four of five Rhesus monkeys that received islet xenotransplantation had graft survival with good glycemic control for more than six months [9,11]. Numerous trials of xenotransplantation in non-human primates were also conducted, with success in curing hyperglycemia and lowering daily insulin dose requirements [9]. Unfortunately, due to venous thromboembolic consequences, the therapeutic advancement of specifically targeted antibodies was not further investigated [11].

A known consequence of xenotransplantation via the portal vein is an immediate blood-mediated inflammatory reaction (IBMIR), in which approximately 70% of islet cells are lost [9,11,16]. This significant loss of islets immediately after transplantation occurs via a series of reactions that include activation of the complement system, activation of intrinsic and extrinsic coagulation pathways, activation of platelets, and infiltration of inflammatory cells, particularly neutrophils and monocytes [9,16]. Novel strategic methods targeting IBMIR are under investigation to minimize islet cell loss and prolong graft survival [11,16].

Latest innovations in swine genetic alteration and immune suppression regimes in preclinical pig to non-human primate islet cell xenotransplantation research have resulted in additional understanding into the etiology of islet cell rejection as well as the advancement of effective pancreatic islet cell xenotransplantation [9]. In addition to genetic modification of porcine islets and proper therapeutic immunosuppressive therapy against xenografts [13], tolerance induction would be especially crucial following transplantation to control blood glucose levels [8,15,16]. The induction of tolerance may have a greater beneficial impact, with the ability to promote tolerance to the grafted porcine islet as well as to avoid recurrent episodes of autoimmune defense mechanisms [16]. To prolong islet transplants, bone marrow infusions have been used to promote stable hematopoietic cell chimerism [16,22]. Indeed, though PERV transmission remains a major limiting factor for successful xenotransplantation, even after a lengthy monitoring period, there has been no proof of zoonosis detected in patients with type 1 diabetes mellitus who underwent swine islet cell transplants [9].

## Conclusions

Xenotransplantation holds a promising future for many patients, especially those with end-stage renal disease or absurd serum glucose levels. The ideal porcine candidate for xenotransplantation is a pig devoid of any zoonotic micro-organisms and PERV-C. PERV-inactivation has proved to be beneficial for successful xenotransplantation. Pigs that have been genetically modified to PERV have the potential to offer safe and effective organ and tissue supplies for xenotransplantation in the future. Combating zoonotic micro-organisms can be accomplished through evaluation and screening processes and by breeding genes devoid of PERV, resulting in PERV knockout pigs, both of which may aid in reducing the risk of zoonotic infections. Pre-xenotransplantation microbial investigations, monitoring of the recipient, and a treatment plan designed especially for the recipient to avoid disease conditions all require careful planning. The latest innovations in xenotransplantation need to be built upon by evaluating the limitations of genetic engineering and proposing strategies for their advancement. Still, significant challenges to clinical human islet xenotransplantation are present, and future efforts should be directed toward developing an ideal genetically engineered swine islet donor source and establishing long-term viable islets with clinically meaningful immunosuppression. Researchers have proven that by successfully bypassing all the hurdles faced in xenotransplantation, in addition to being an effective means of obtaining normal blood glucose levels and reducing several illnesses associated with diabetes, it might embark a novel way for new therapeutic options for type 1 diabetic patients. Lowering the risk of post-xenotransplantation rejection reactions, IBMIR loss, acute inflammation, and chronic inflammatory markers in newly genetically modified porcines will not only increase graft survival but also further advance this field of research.
